# Functional Genomics, Transcriptomics, and Proteomics Reveal Distinct Combat Strategies Between Lineages of Wood-Degrading Fungi With Redundant Wood Decay Mechanisms

**DOI:** 10.3389/fmicb.2020.01646

**Published:** 2020-07-28

**Authors:** Gerald N. Presley, Jiwei Zhang, Samuel O. Purvine, Jonathan S. Schilling

**Affiliations:** ^1^Department of Wood Science and Engineering, Oregon State University, Corvallis, OR, United States; ^2^Department of Bioproducts and Biosystems Engineering, University of Minnesota, Saint Paul, MN, United States; ^3^Environmental Molecular Sciences Laboratory, Pacific Northwest National Laboratory, Richland, WA, United States; ^4^Department of Plant and Microbial Biology, University of Minnesota, Saint Paul, MN, United States

**Keywords:** fungal interactions, secondary metabolites, brown rot, microbial ecology, wood decay basidiomycetes

## Abstract

Wood-degrading fungi vary in their strategies for deconstructing wood, and their competitive successes shape the rate and fate of carbon released from wood, Earth’s largest pool of aboveground terrestrial carbon. In this study, one-on-one interspecific interactions between two model brown rot (carbohydrate-selective) fungi, *Gloeophyllum trabeum* and *Rhodonia* (*Postia*) *placenta*, were studied on wood wafers where a clearly resolved interaction zone (IZ) could be generated, reproducibly. Comparative RNAseq and proteomics between the IZ and non-interacting hyphae of each species identified combative strategies for each fungus. Glycoside hydrolases were a relatively smaller portion of the interaction secretome compared to non-interacting hyphae. The interaction zone showed higher pectinase specific activity than all other sampling locations, and higher laminarinase specific activity (branched β-glucan proxy) was seen in the IZ secretome relative to equivalent hyphae in single-species cultures. Our efforts also identified two distinct competitive strategies in these two fungi with a shared nutritional mode (brown rot) but polyphyletic ancestral lineages. *Gloeophyllum trabeum* (Gloeophyllum clade) upregulated more secondary metabolite (SM) synthesis genes in response to a competitor than did *R. placenta*. *R. placenta* (Antrodia clade) upregulated a larger variety of uncharacterized oxidoreductases in interacting hyphae, suggesting that these may play a role in mediating competitor response in this fungus. Both species produced several hypothetical proteins exclusively in the interaction zone, leaving questions as to the function of these proteins. This work supports the existence of multiple interaction strategies among brown rot fungi and highlights the functional diversity among wood decay fungi.

## Introduction

Wood-degrading basidiomycetes live in complex microbial communities, sometimes with thousands of other fungal species in a single piece of degrading wood (Rajala et al., 2012). These fungi utilize a variety of combative strategies to compete for resources which impact wood decomposition rates and help modulate global carbon cycles (Hiscox et al., 2015; O’Leary et al., 2018). The physical, biochemical, and chemical responses basidiomycetes produce when confronted with competitors can be replicated in synthetic culture and used to study the mechanistic basis of these interactions (Boddy, 2000). Lab-based study of fungal interspecific interactions can enable a better understanding of fundamental processes that drive forest ecosystem function.

One component of competitor response is the expression of fungal cell wall-degrading enzymes. Fungal cell walls, composed primarily of chitin and branched beta glucans, can serve as a source of nutrients for competing fungi as the “losing” competitor’s territory is overtaken (Boddy and Hiscox, 2016). Cell wall beta-glucans and chitin are degraded by extracellular glycoside hydrolases (GHs) belonging to several GH families including GH 16 β-glucanases, GH 18 chitinases, and GH 20 β-N-acetyl-glucosaminidases (Martin et al., 2007; Langner and Gohre, 2016). Elevated GH 18 chitinase expression levels have been shown in secondary colonization of dead fungal hyphae of *Heterobasidion irregulare* by the white rot basidiomycete *Phaneorchaete chrysosporium*, a pattern which may also appear in response to the hyphae of living fungal competitors (Karlsson et al., 2016). This type of response is likely widespread among wood-degrading saprophytes, as hyphal displacement is a common process in natural successional cycles among decay fungi (O’Leary et al., 2018). In contrast, expression levels of other classes of GHs involved in plant cell wall degradation have been shown to remain constant during interspecific interactions, as seen in the white rot fungus *Pycnoporus coccineus* paired against *Coniophora puteana* or *Botrytis cinerea* (Arfi et al., 2013). This pattern, albeit produced from a combination of model species, suggests a diversion of resources away from plant cell wall metabolism and toward fungal cell wall metabolism during interspecific combat.

Basidiomycete oxidoreductases known to participate in lignin decomposition also appear to play a role in mediating interspecific interactions (Hiscox and Boddy, 2017). Laccase activity is widely induced in interspecific interactions of white rot basidiomycetes (Baldrian, 2004; Snajdr et al., 2011) as are ligninolytic peroxidases (Hiscox et al., 2010) and the expression of uncharacterized oxidoreductases (Eyre et al., 2010). The biological function of these enzymes in interactions is not known, but upregulated laccases may be involved in melanin synthesis at the interaction zone, which can serve as a protective barrier between interacting fungi (Silar, 2005; Kuees and Ruehl, 2011; Cordero and Casadevall, 2017). Laccases can also degrade toxic metabolites produced by competitors during interactions (Hiscox and Boddy, 2017), which is an important biological function in interactions laden with antimicrobial metabolites.

In addition to a rich enzymatic diversity, saprotrophic basidiomycetes are also rich sources of secondary metabolites (SMs), and possess a variety of SM-synthesizing genes in their genomes (Quin et al., 2013; Riley et al., 2014). The biological functions of most basidiomycete SM-synthesizing genes are predominantly unknown but could plausibly function as antibiotics against competitor species. Several types of SMs including terpenes and polyketide-derived molecules are produced in response to fungal competitors (Hynes et al., 2007; Evans et al., 2008; El Ariebi et al., 2016; Yao et al., 2016; Hiscox and Boddy, 2017; O’Leary et al., 2019). *Gloeophyllum* sp. are wood-degrading basidiomycetes known to produce several terpenes such as gloeophyllins (Rasser et al., 2000; Han et al., 2015) and orsellinic acid-based polyketides such as oosponols (Sonnenbichler et al., 1997; Rasser et al., 2000) in axenic culture and during interactions (Sonnenbichler et al., 1993, 1994). *Gloeophyllum trabeum* is a well-studied model brown rot fungus whose genome encodes a several SM synthesis genes predicted to produce SM scaffolds of known *Gloeophyllum* metabolites, but their biological role is unknown (Sonnenbichler et al., 1994; Lackner et al., 2012; Wawrzyn et al., 2012). *G. trabeum* has been studied extensively in axenic culture and is an ideal model brown rot system to study the role of these genes in facilitating basidiomycete interactions.

In this study, we investigated the mechanisms of interaction between two model brown rot fungi, *Gloeophyllum trabeum* and *Rhodonia placenta*, both of which are common inhabitants of softwood lumber and could presumably come into contact with one another in this environment. The interactions were facilitated on aspen wood wafers so functional -omics techniques could be used to compare previous interaction cultures to each individual species (Zhang et al., 2016, 2019). The two fungi were grown against one another to simulate interspecific interactions and secreted proteins and RNA were extracted within and around the interacting hyphae. The proteome composition and plant and fungal polysaccharide-degrading activity was compared to actively growing hyphae of single-species cultures as were gene expression profiles from equivalent locations to allow the overlay of transcriptomics, proteomics, and enzyme activity data. This work identifies several proteins that are important in mediating basidiomycete interspecific interactions and identifies two different interaction strategies in the species tested.

## Materials and Methods

### Culture Conditions and Interaction Microcosms

**FIGURE 1:**
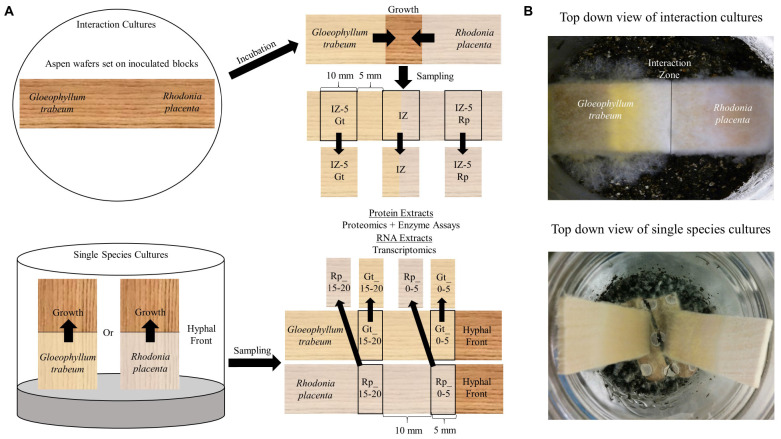
**(A)** Diagrammatic representation of the two culture types and sampling regime used to generate the different types of decayed wood pieces that were extracted for secretomic or transcriptomic analysis. Competition microcosms used to simulate interspecific interactions between *Gloeophyllum trabeum* and *Rhodonia placenta* in this study. Pre-inoculated wood blocks for each species are used as inocula for each end of a thin aspen wafer placed across them. The two species grow together and contact in the middle of the wafer where the interaction zone and surrounding hyphae 5 mm outside of the interaction zone is sampled for protein and RNA for proteomic and transcriptomic analysis. Single species cultures were the same cultures generated in previous studies (Zhang et al., 2016, 2019; Presley et al., 2018). Gene expression data from single species cultures was adapted from Zhang et al. (2016, 2019) while proteomics data from this culture type was adapted from Presley et al. (2018). **(B)** Pictures of the two culture types from the top down.

*Gloeophyllum trabeum* ATCC 11539 and *Rhodonia* (*Postia*) *placenta* MAD 698-R were maintained on malt extract agar and 1 cm diameter agar plugs were used to inoculate soil block microcosms as previously described (Presley and Schilling, 2017). These test strains were chosen for the depth of experimental data associated with them and because they grew at similar rates in the experimental setup used here, which enabled reproducible interactions to be generated. Sterile 19 mm aspen blocks were degraded for 4 weeks in soil block jars for each species and then were used as inoculum for interaction microcosms. Interactions were simulated by placing one *G. trabeum* and one *R. placenta* block opposing one another in empty soil microcosms and laying a 60 × 23 × 3 mm aspen wafer (largest face in cross section) across the top of the two blocks (Figure 1). Fungal hyphae grew together until they met in the center of the wafer, forming an interaction zone which was then sampled along with surrounding hyphae for protein and RNA, as described below.

### Protein Extraction and Purification

Interaction wafers were sampled for protein at three sections, one consisting a 10-mm section surrounding the interacting hyphae (IZ), and one 10 mm section 5 mm behind the boundary of the interaction zone toward each species (IZ-5Rp and IZ-5Gt). Non-degraded aspen wood was extracted as a control. For protein extracts, three replicate pools of 15 interaction wafers per poor were extracted with 100 ml of cold extraction buffer (0.5 M NaCl, 0.05 acetate, 0.05% tween 80, pH 5.0) for 24 h at 4°C with gentle shaking. Extracts were filtered through polyester cloth, centrifuged at 4000 × g for 30 min at 4°C, and filtered through 0.2 μm sterile PVDF filters. Extracts were exchanged into 0.05 M citrate pH 5.0 and concentrated using Vivaspin Polyethersulfone (PES) 10 kDa cutoff membranes prior to freezing at −20°C. Protein concentrations of extracts were determined using a BioRad protein assay kit (Hercules, CA, United States).

### Biochemical Assays

Protein extracts were assayed for poly, oligo, and disaccharide-degrading activity and compared to previously generated values for single-species cultures on aspen wood 0–5 mm from the advancing hyphal front (Rp/Gt_0–5) and 15–20 mm behind the hyphal front (Rp/Gt_15–20) (Presley et al., 2018). Endoglucanase (EG), xylanase (Xyl), laminarinase (Lam), and pectinase (Pec) activity were measured in triplicate using the DNS assay for reducing sugars (Ghose, 1987). Reactions were done at 50°C in 0.05 M citrate at pH 5.0 using 1.5% carboxymethyl cellulose (EG), 2% birchwood xylan (Xyl), 0.25% laminarin (Lam), and 0.5% polygalacturonic acid (Pec) as substrates. Color was developed at 90°C and absorbance of developed reactions was measured at 540 nm. Units were defined as the amount of enzyme required to liberate 1 μmol of glucose, xylose, glucose, or galacturonic acid reducing equivalents per minute under the above conditions for EG, Xyl, Lam, and Pec activity, respectively.

β-glucosidase (BGL) and β-N-acetylglucosaminidase (BNAG) were measured in triplicate by monitoring the liberation of 4-nitrophenol from 4-nitrophenyl-β-D-glucoside and 4-nitrophenyl-β-D-N-acetylglucosamine, respectively. Reactions were carried out in 10 mM of substrate and 0.05 M citrate, pH 5.0 at 50°C and quenched with 2 volumes of 0.2 M Na_2_CO_3_. Absorbance at 400 nm of quenched reactions was measured and one unit of activity was defined as the amount of enzyme required to liberate 1 μmol of 4NP per minute in the above conditions.

### Mass Spectrometry

Portions of the same protein extracts used for biochemical assays were reserved for proteomics analysis after extraction and purification, as above. Three separate extracts of pools of 15 interaction wafers per pool were analyzed for proteomic studies. Extracts were precipitated in TCA/acetone, pelleted, and reconstituted in saturated guanidine-HCl. The protein concentration of each cellular extract was determined by BCA assay (Smith et al., 1985). For each sample, processing replicates were performed; 50 μg of protein was aliquoted to low-retention Eppendorf tubes for downstream sample processing. All samples were incubated for 30 min at 60°C with tris(2-carboxyethyl)phosphine (Bond-Breaker TCEP, Thermo Fisher Scientific, Rockford, IL, United States) to reduce disulfide bonds. Alkylation of cysteine residues was performed by treatment with 50 mM iodoacetamide, which was added from a 500 mM iodoacetamide, 500 mM ammonium bicarbonate stock solution. After addition of iodoacetamide, all samples were incubated at room temperature in the dark for 40 min on a rocker. Each sample was then diluted to 0.9 M urea with 500 mM ammonium bicarbonate. To each 50 μg sample, 0.1 μg of mass spectrometry grade trypsin (Promega Corp., Madison, WI, United States) was added and incubated overnight at 37°C. Peptides were extracted from each sample using solid phase extraction with Discovery C18 50 mg resin columns (Supelco, St. Louis, MO, United States). Each column was activated with 2 ml of methanol followed by equilibration with 6 ml of 18 mΩ water. The sample was applied to the column and then the column was washed with 8 ml of 50 mM ammonium bicarbonate. Peptides were eluted with two 0.9 ml washes of 40% acetonitrile. Samples were dried using a centrifugal concentrator (Thermo Fisher Scientific, Asheville, NC, United States) and stored at −20°C until LC/MS analysis.

Peptides were solubilized in 150 μl solvent A (0.1% formic acid). For LC/MS analysis, 5 μl of sample was injected onto a Jupiter C18 resin reverse-phase column (3 μm particle size, 35 cm long, 75 μm inner diameter; Phenomenex, Torrance, CA, United States). The peptides were eluted at 0.3 μl min^–1^ with an Agilent (Santa Clara, CA, United States) 1200 high-performance liquid chromatograph with solutions of solvent A and 0.1% formic acid in acetonitrile (solvent B) using the following conditions: 0–30 min, isocratic at 100% solvent A; 30–32 min, linear gradient to 8% solvent B; 32–50 min, linear gradient to 12% solvent B; 50–105 min, linear gradient to 35% solvent B; 105–127 min, linear gradient to 60% solvent B; 127–130 min, linear gradient to 95% solvent B; and isocratic at 95% solvent B for 5 min. Eluted peptides were introduced into an Orbitrap XL mass spectrometer (Thermo Fisher Scientific, Waltham, MA, United States) by electrospray ionization.

Spectra were collected in a data-dependent mode, with the five most intense ions in each survey scan selected for collisional-induced dissociation in the five subsequent scans. Spectra were deconvoluted using the DeconMSn software (Mayampurath et al., 2008) to more accurately assign parent ion mass and ion charge state. Spectra were then searched against predicted peptides derived from the fungal genome sequences via MS-GF+ [software used to analyze tandem mass spectra data, (Kim and Pevzner, 2014)], using a 20 ppm parent ion mass tolerance in searches of tryptic peptides with a variable post-translational modification of oxidized methionine. Peptides from the interaction zone were searched against predicted peptides from each fungus in a single concatenated FASTA file. A Q value cut off (≤0.01) was utilized to allow a ∼1% false discovery rate (FDR) at each individual data set level, as assessed from a decoy identification search utilizing the reverse fungal genome sequence. Twenty-one peptides mapped to more than one protein in the interaction zone, however, none of these had hits to proteins that were in the opposing species. Spectra that mapped to more than one protein were assigned to the first occurrence of a matching protein in the concatenated FASTA file. The raw proteomics data is deposited in the PRIDE/ProteomeXchange database, project accession: PXD009480 and project doi: 10.6019/PXD009480.

### RNA Extraction and RNA-Seq

Five mm sections from equivalent locations on two replicate wood wafers (about 200 mg each) were snap frozen in liquid nitrogen and ground to powder prior to extraction in 2 ml of TRIzol (Life Technologies). Samples were taken from locations equivalent to those used for proteomics analysis. Samples were purified using an RNeasy minikit (Qiagen, Inc.) with on-column DNase digestion. RNA quality was monitored using an Agilent Bioanalyzer (Agilent Technologies) and only RNA samples with RNA integrity >8 were used for RNA-seq. RNA-seq was performed as previously described (Zhang et al., 2016) on a HiSeq 2500 system (Illumina) at the University of Minnesota Genomics Center. Duplicate samples of each type, interaction zone (IZ), 5 mm outside of the interaction toward *G. trabeum* (IZ-5Gt), and 5 mm outside of the interaction toward *R. place*nta (IZ-5Rp) were sequenced.

RNA-seq data analysis was performed on the Galaxy platform^1^ using described procedures for differential expression analysis (Trapnell et al., 2012). Raw reads were cleaned with Trimmomatic (v0.3) and mapped against *R. placenta* MAD698-R (v1.0) and *G. trabeum* ATCC11539 (v1.0) genomes together in a single concatenated file using TopHat (v2.0.13). Previously generated RNA-seq data from single-species cultures of both species grown on equivalent on aspen wafers were used for comparison to data generated in this study (Zhang et al., 2016, 2019). Expression levels at the leading hyphal edge of fungal cultures on aspen and 5 mm outside of the interaction zone from hyphae of each of the interacting species were compared to expression levels found at the IZ. Reference transcript models from the JGI database were used to determine differences in expression levels as Reads Per Kilobase Per Million (RPKM) in pairwise comparisons among samples using Cuffdiff (Galaxy Tool Version 2.2.1.3) using geometric normalization. FDR was set at <0.05. Transcripts with fourfold greater RPKM values in the IZ sample relative to other samples were identified. When the IZ RPKM values were compared to either the *G. trabeum*-only samples or the *R. placenta*-only samples only the reads from the IZ that mapped to the *G. trabeum* or *R. placenta* genome, respectively, were considered for fold-change analysis. Most reads from *G. trabeum* or *R. placenta*-only samples did not map (RPKM = 0) to the opposing fungus’s genome, and those that did had RPKM values generally well below 1. The raw RPKM sequence data can be found in Supplementary Table S1 and in the Gene Expression Omnibus database (GSE151023).

### Secondary Metabolite Synthesis Gene Expression Analysis and Cluster Identification

Basidiomycete secondary metabolite (SM) scaffold-synthesizing genes including polyketide synthases (PKSs) (Lackner et al., 2012), sesquiterpene synthases (STSs) (Wawrzyn et al., 2012), non-ribosomal peptide synthases (NRPSs) (Kalb et al., 2013), and NRPS-like reductases (AFRs) (Brandenburger et al., 2016) were identified in the *G. trabeum* and *R. placenta* genomes. SM synthesis gene clusters analysis was performed with anti-smash fungal version (Weber et al., 2015) and gene clusters of upregulated SM genes were identified. Relative expression levels of genes in SM clusters of interest in the IZ were determined relative to levels previously found at the hyphal front of single-species cultures (Zhang et al., 2016, 2019).

### Sequence Analysis

Orthologous proteins were identified among those identified in secretomes and among genes fourfold upregulated in the interaction microcosms using the TRIBE-MCL clustering method (Enright et al., 2002). Secretion signal peptides for upregulated and secretome proteins were identified using Signal P prediction server (Armenteros et al., 2019).

## Results

### Generation of Interaction Microcosms

*Rhodonia placent*a and *Gloeophyllum trabeum* both colonized interaction aspen wafers at similar rates. Once established on the portion of the wafers in contact with the inoculated blocks, in most cases the hyphae advanced on to uncolonized wood in the direction of the opposing fungus and met in the wafer center within 2 weeks. While the location of contact varied somewhat from microcosm to microcosm, the microcosm design was reproducible enough to generate 47 wafers to sample (3 sets of 15 wafers for proteomics and 2 for RNAseq). Interaction outcomes were not measured because the cultures were sampled just as the hyphae came together. However, preliminary observations of longer-incubated cultures indicated that deadlock was the most common outcome under these conditions.

### Secretome Composition of Interacting Hyphae Compared to Single-Species Cultures

**FIGURE 2:**
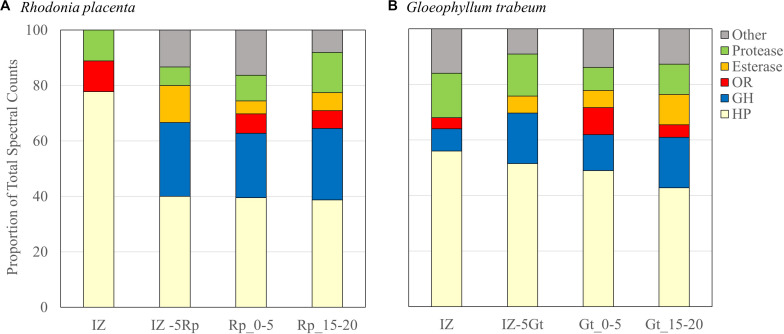
Secretome compositions from *Rhodonia placenta* **(A)** and *Gloeophyllum trabeum* **(B)** isolated from interacting (IZ) and non-interaction interacting hyphae 5 mm outside of the interaction zone (IZ-5Rp/Gt), and 0–5 mm (Rp/Gt_0–5), and 15–20 mm (Rp/Gt_15–20) behind the hyphal front of single species cultures. Protein categories are presented as proportions of the total numbers of spectral counts. HP, hypothetical protein; GH, glycoside hydrolase; OR, oxidoreductase.

Secreted proteins isolated from the IZ were compared to those from non-interacting hyphae 5 mm outside of the interaction zone for each species (IZ-5Rp/Gt) and extracts from single-species cultures 0–5 mm (Rp/Gt_0–5) and 15–20 mm (Rp/Gt_15–20) behind an actively growing hyphal front (Presley et al., 2018). Interaction microcosms showed a reduced diversity of secreted protein compared to single-species cultures. Peptides from only 36 different proteins were identified in the IZ, 10 from *R. placenta* and 26 from *G. trabeum*, compared to 42 and 194 proteins identified in Rp_0–5 and Gt_0–5 extracts, respectively. *G. trabeum* proteins were responsible for 74.3% of the spectral counts observed in interactions while *R. placenta* proteins accounted for 25.7%. Fifteen and thirty-three proteins were identified in IZ-5Rp and IZ-5Gt extracts, respectively, whereas equivalent sections in single species cultures of *R. placenta* (Rp_15–20) and *G. trabeum* (Gt_15–20) had 62 and 110 identified proteins, respectively. Glycoside hydrolases (GHs) as a proportion of all proteins identified were lower in IZ extracts (6%) than in extracts outside of the interaction zone (IZ-5Rp/Gt, 21%). All single-species culture extracts had a higher proportion of glycoside hydrolases in than the IZ (15–21%) (Figure 2; Presley et al., 2018). Although GH production in the IZ was relatively lower than other extracts, a GH 115 putative α-glucuronidase not found in single-species cultures was produced outside (*G. trabeum* and *R. placenta*) and within (*G. trabeum*) the interaction zone (Supplementary Tables S1–S3). One of these (Gt 121308) was produced in particularly high abundance and it constituted 20% of all protein observations of proteins in the IZ-5Gt extracts.

No esterases/lipases were identified in extracts of the IZ while this group of enzymes composed 8% and 6–9% of the proteins found in extracts outside the interaction zone and in single-species cultures, respectively. Extracts from the interaction zone were primarily composed of hypothetical proteins (62%), compared to 48% and 41–47% in non-interacting hyphae and single-species cultures, respectively. Several proteins, eight from *R. placenta* and seven from *G. trabeum*, were found exclusively in the IZ (Table 1). The majority of these were uncharacterized hypothetical proteins, but both fungi produced their own putative acid protease exclusive to the interaction zone, both of which contained secretion signals predicted by SignalP (Armenteros et al., 2019).

**TABLE 1:** Proteins found exclusively in the interaction zone (IZ) produced by *Gloeophyllum trabeum* and *Rhodonia placenta*.

**Protein ID^a^**	**Name**	**Putative function^b^**	**Observations^c^**	**Size (kDa)**	**Signal P^d^**
**Rhodonia placenta**					
105721	FAD-binding protein	FAD-binding protein	4	53.28	18–19
1183565	Hypothetical protein	No strong hits	2	40.45	No
92184	Hypothetical protein	No strong hits	1	85.01	No
99255	Hypothetical protein	No strong hits	7	82.27	No
93455	Hypothetical protein	No strong hits	1	14.97	No
98779	Hypothetical protein	No strong hits	11	79.06	No
96562	Hypothtical protein	No strong hits	36	52.89	No
1127001	Protease, Acid	Polyporopepsin	3	42.22	19–20
***Gloeophyllum trabeum***					
101772	Hypothetical protein	No strong hits	1	28.76	19–20
91843	Hypothetical protein	Pumilo domain	1	137.06	No
128123	Hypothetical protein	No strong hits	1	22.38	No
108762	Hypothetical protein	No strong hits	7	17.55	17–18
112815	Hypothetical protein	No strong hits	1	32.95	No
136352	Protease, Acid	Acid protease	4	107.7	19–20
81033	Ser/Thr Protein Phosphatase	Ser/Thr Protein Phosphatase	1	66.63	No

**^*a*^Protein ID numbers from the DOE JGI Mycocosm database (Grigoriev et al., 2014). ^*b*^Putative functions determined by BLAST searches of SWISS PROT database (Bateman et al., 2015). ^*c*^Number of observations in the interaction zone protein sample (spectral counts). ^*d*^Signal peptides identified with Signal P 5.0 (Armenteros et al., 2019).**

### Enzyme Activities

**FIGURE 3:**
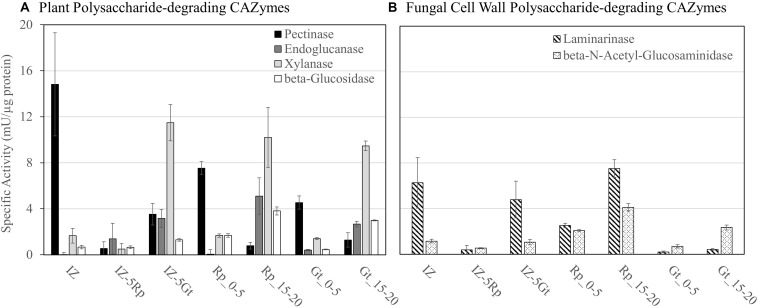
Enzyme specific activities for **(A)** plant polysaccharide-degrading CAZymes and **(B)** fungal cell wall polysaccharide-degrading enzymes in protein extracts from interacting (IZ) and non-interacting hyphae 5 mm outside of the interaction zone (IZ-5Rp/Gt), and 0–5 mm (Gt/Rp_0–5) and 15–20 mm (Rp/Gt_15–20) behind the advancing hyphal front in single species cultures.

Specific activities for six enzymes, pectinase (Pec), endoglucanase (EG), xylanase (Xyl), β-glucosidase (BGL), Laminarinase (Lam), and β-N-acetylglucosaminidase (BNAG), were measured on protein extracts from the IZ and outside of the interactions zone (IZ-5Rp/Gt) and were compared to those measured in single-species cultures 0–5 mm (Rp/Gt_0–5) or 15–20 mm (Rp/Gt_15–20) from the advancing hyphal front (Figure 3). Extracts of the IZ showed the highest pectinase specific activity, twofold and threefold higher than those found at Rp_0–5 mm and Gt_0–5, respectively. Laminarinase specific activity was also significantly higher (*p* < 0.05, Tukey’s HSD) in IZ extracts than all others except IZ-5Gt and Rp_15–20 mm. Only two GHs, both produced by *G. trabeum*, in the IZ, a GH 115 (121308) and a GH 3 (69843) were identified and neither of these are likely endo-laminarinases. All other measured activities were generally low in IZ extracts, but some plant polysaccharide-degrading activities increased to levels equivalent to those found in single-species cultures or IZ-5Rp/Gt extracts (Figure 3). The GH 3 produced in non-interacting *R. placenta* cultures was orthologous to those produced in both interacting and non-interacting *G. trabeum* secretomes, suggesting they have a similar function in both species (Supplementary Data S2).

### Transcriptional Profiling of Interacting Hyphae

**FIGURE 4:**
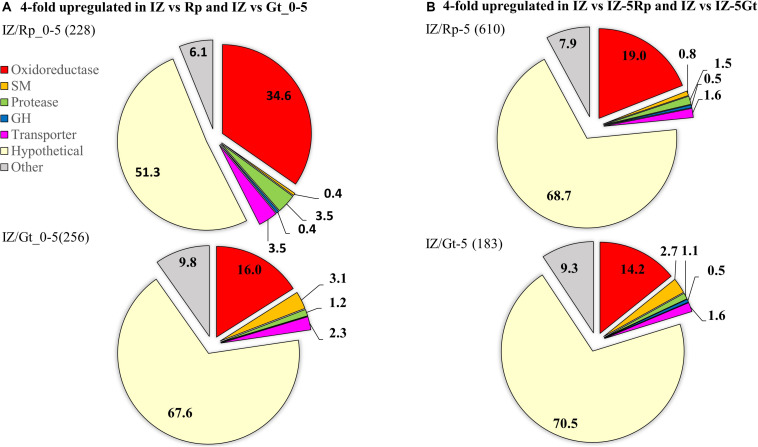
Genes fourfold upregulated in interacting hyphae (IZ) compared to non-interacting hyphae **(A)** at the leading hyphal front of single species cultures (Rp/Gt_0–5) and **(B)** outside of the interaction zone (IZ-5Rp/Gt) of *Rhodonia placenta* and *Gloeophyllum trabeum*. Categories are represented as proportions of all genes fourfold upregulated and the total number of genes upregulated are in parentheses. SM, secondary metabolite scaffold-synthesizing genes; GH, glycoside hydrolase.

RNA-seq was used to compare gene expression levels in the IZ to surrounding hyphae and single-species cultures for each species. In the interaction zone, 65.5% of the reads mapped to the *G. trabeum* genome, while 36.5% mapped to the *R. placenta* genome. Of the genes that are upregulated at least fourfold in the IZ relative to Rp_0–5 and Gt_0–5, 34.6% (79) and 16.0% (41) were oxidoreductases (ORs), respectively, indicating a significant role for this enzyme group in the IZ (Figure 4A). *G. trabeum* upregulated a greater number of secondary metabolite (SM) synthesizing genes (eight) at least fourfold in the interaction zone relative to Gt_0–5 than *R. placenta* did relative to Rp_0–5 (one). *R. placenta* upregulated more proteases at least fourfold in the interaction zone relative to Rp_0–5 (eight) than *G. trabeum* did relative to Gt_0–5 (three) (Figure 4A). Similar patterns were seen when gene expression levels in the interaction zone were compared to non-interacting hyphae around it (IZ-5Rp/Gt). Of the genes that are fourfold upregulated in the interaction zone relative to IZ-5Rp and IZ-5Gt, 19.0% (116) and 14.2% (26) were putative oxidoreductases, respectively (Figure 4B).

The 25 most highly upregulated genes in the interaction zone relative to the *R. placenta* hyphal front (Rp_0–5) included 5 hypothetical proteins, 14 uncharacterized ORs including putative cytochrome p450s, an aryl alcohol dehydrogenase and a protease (Table 2). One of these, Rp105721 a putative FAD-binding protein, was also identified in interaction secretomes and it also had no orthologs among upregulated genes in *G. trabeum*. Of the 25 most highly upregulated genes in the interaction zone relative to Gt_0–5, the majority (17) were hypothetical proteins. Four of these hypothetical proteins, Gt101772, 44870, 92695, and 129354 were also found in the interaction secretomes. The 25 most highly upregulated genes also included a laccase, an oxalate decarboxylase, an O-methyl transferase, a polyketide synthase, and a hydrophobin (Table 2). Only a few GHs were upregulated in the interaction zone vs. non-interacting hyphae for *R. placenta* (4) or *G. trabuem* (1). In the interaction zone, both species upregulated a GH 16 protein and *R. placenta* also upregulated two GH 18 chitinase domain-containing proteins, as well as a GH 5 β-1,3-glucanase. Hypothetical proteins were the largest category of upregulated proteins among all comparisons, ranging from 51.3 to 70.5% of all genes upregulated at least fourfold in the interaction zone (Figure 4). This is likely not due to any specific enrichment in hypothetical proteins in the interaction, but probably due to the general high abundance of hypothetical proteins in basidiomycete genomes.

**TABLE 2:** Top 25 genes most highly upregulated in interacting hyphae (IZ) compared to the leading hyphal edge (Rp/Gt_0–5) of single-species cultures of *Gloeophyllum trabeum* (Gt) and *Rhodonia placenta* (Rp).

**Most upregulated in IZ/Gt_0–5**						**Most upregulated in IZ/Rp_0–5**					
**Protein ID^a^**	**Name^b^**	**RPKM IZ**	**RPKM Gt_0–5^c^**	**Log_2_ IZ/Gt_0–5**	**Signal P^d^**	**Protein ID^a^**	**Name^b^**	**RPKM IZ**	**RPKM Rp_0–5^e^**	**Log_2_ IZ/Rp_0–5**	**Signal P^d^**
137245	Hypothetical protein	492.6	0.4	10.1	No	63488	Oxidoreductase	211.1	0.7	8.3	No
92695	Hypothetical protein	3599.5	8.8	8.7	19–20	60565	Oxidoreductase	129.4	0.5	8.0	No
103366	Oxalate decarboxylase	463.5	1.5	8.2	19–20	105320	Hypothetical protein	65.6	0.3	7.9	22–23
44870	Hypothetical protein	721.6	3.9	7.5	21–22	105721	Oxidoreductase	257.9	1.5	7.4	No
48004	Vacuolar protein	14.4	0.1	7.4	No	47260	Oxidoreductase	389.2	2.6	7.2	No
95418	Hypothetical protein	203.6	1.4	7.2	15–16	35307	Oxidoreductase	545.3	4.3	7.0	No
134059	Hypothetical protein	5481.2	69.5	6.3	23–24	54275	Hypothetical protein	94.8	1.0	6.6	20–21
95488	Hypothetical protein	130.7	1.8	6.2	No	48020	Cytochrome p450	154.3	1.8	6.4	No
114641	O-methyl transferase	3051.3	45.2	6.1	No	45365	Uncharacterized transporter	49.1	0.8	5.9	No
134822	Hypothetical protein	404.2	6.3	6.0	19–20	126377	Oxidoreductase	156.9	2.9	5.8	No
104526	Hypothetical protein	245.2	3.9	6.0	19–20	92340	Cytochrome p450	77.6	1.5	5.7	27–28
96525	Hypothetical protein	159.9	2.8	5.8	No	46594	Aryl alcohol dehydrogenase	79.3	1.5	5.7	No
104708	Oxidoreductase	741.1	13.8	5.7	No	116256	Potassium channel	45.0	0.9	5.7	No
112738	Hypothetical protein	500.8	9.4	5.7	No	126381	Protease, acid	82.2	1.6	5.6	26–27
129354	Hypothetical protein	587.6	11.3	5.7	19–20	105719	Cytochrome p450	68.3	1.6	5.5	27–28
113399	Oxidoreductase	840.4	16.9	5.6	No	106360	Hypothetical protein	69.5	1.7	5.4	No
45373	Hypothetical protein	98.3	2.0	5.6	21–22	103283	Expansin-like protein	25.3	0.6	5.3	20–21
67066	Hypothetical protein	1826.5	37.4	5.6	No	99672	Oxidoreductase	31.1	0.8	5.2	No
94357	Hypothetical protein	58.7	1.2	5.6	17–18	100325	Hypothetical protein	150.3	4.0	5.2	20–21
50822	Polyketide synthase I	184.0	4.0	5.5	No	63451	Oxidoreductase	67.6	2.0	5.1	No
19724	Hydrophobin	2085.5	46.0	5.5	No	45316	Hypothetical protein	121.7	4.1	4.9	No
50761	Hypothetical protein	695.6	15.5	5.5	No	61173	Oxidoreductase	188.3	6.4	4.9	No
101772	Hypothetical protein	372.3	8.8	5.4	25–26	91006	Alcohol dehydrogenase	125.1	4.3	4.9	No
130426	Laccase 2	47.0	1.1	5.4	20–21	115141	Lipase	204.6	7.2	4.8	26–27
17704	Hypothetical protein	149.8	3.8	5.3	No	45371	Oxidoreductase	33.2	1.2	4.8	No

**RPKM values for each gene at each condition is shown with the log2 of the ratio of IZ. ^*a*^Protein ID numbers from the DOE JGI Mycocosm database (Grigoriev et al., 2014). ^*b*^Putative functions determined by BLAST searches of SWISS PROT database (Bateman et al., 2015). ^*c*^Gt_0–5 RPKM value adapted from Zhang et al. (2019). ^*d*^Secretion signals for detected protein sequences were detected using Signal P algorithm (Armenteros et al., 2019). ^*e*^Rp_0–5 RPKM values adapted from Zhang et al. (2016).**

### Secondary Metabolite Synthesizing Genes in *G. trabeum*

**FIGURE 5:**
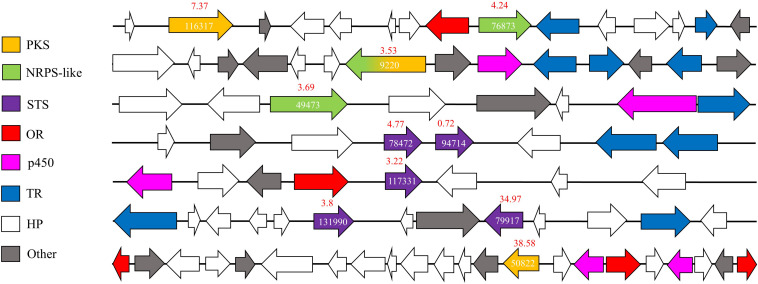
Secondary metabolite (SM) synthesis clusters in *Gloeophyllum trabeum* that contain at least one SM-synthesizing gene that is upregulated in interacting hyphae relative to 0–5 mm behind the leading hyphal front of single species cultures. Arrows represent genes with JGI protein ID number over relevant genes. Fold changes in expression levels (IZ/Gt_0–5) are shown in red over each SM synthesis-related gene. JGI protein ID numbers are shown on each annotated gene on the secondary metabolite synthesis cluster. PKS, Polyketide synthases; OR, oxidoreductases; p450, Cytochrome p450; STS, sesquiterpene synthases; NRPS, Non-ribosomal peptide synthase; TR, Transporter.

*G. trabeum* upregulated several SM-synthesizing genes in the interaction zone whereas *R. placenta* upregulated fewer members of this group in the same sample. Proteins upregulated in the interaction zone by *G. trabeum* included two polyketide synthases (PKSs), four sesquiterpene synthases (STSs), one non-ribosomal peptide synthases (NRPSs)-PKS hybrid, and two NRPS-like proteins (Figure 5). AntiSMASH (Weber et al., 2015) SM cluster analysis identified seven gene clusters in which these nine upregulated proteins are located in the *G. trabeum* genome and most are located near cytochrome p450s, other ORs, and/or membrane transporters. One of the PKSs, Gt116317, shares high homology with characterized basidiomycete orsellinic acid synthases (Lackner et al., 2012). Upregulated sesquiterpene cyclases share greatest homology with protoilludene synthases (Gt117331 and Gt78472) and sativene synthases (Gt131990 and Gt79917).

## Discussion

*Rhodonia placenta* and *Gloeophyllum trabeum* are two model brown rot fungi commonly found on softwood lumber and while this interaction may not be likely without human intervention, it is presumed to occur in lumber. The interactions generated in this study were also generated on aspen wood, which is not a native or preferred substrate for these fungi (Krah et al., 2018). However, aspen wood is a widely used model substrate for these two fungi and it was used here to maintain continuity with previous work that was used as a single species control for this study (Zhang et al., 2016, 2019; Presley et al., 2018). The single species cultures used here were also used in a different orientation than the interaction microcosms, which may have caused some differences in gene expression and protein secretion and must be considered when interpreting the results.

Wood-degrading basidiomycete fungi have several tactics to deal with competitors in wood, each characterized by specific morphological and/or biochemical alterations to fungal cultures (Boddy and Hiscox, 2016). This study identifies characteristic differences in protein secretion and gene expression in response to a fungal competitor in the two model brown rot fungi, *Rhodonia placenta* and *Gloeophyllum trabeum*. Secretomes of interacting hyphae showed a general reduction in the prevalence of common decay-related genes such as glycoside hydrolases (GHs) and carbohydrate esterases (CEs) compared to non-interacting hyphae and single-species cultures (Figure 2 and Supplementary Tables S1–S7; Presley et al., 2018). Most of the GHs and CEs previously shown to be secreted by these two fungi in single-species cultures were either downregulated or not significantly changed in the interaction zone (Supplementary Data S1) compared to non-interacting hyphae or single-species cultures. This is similar to the white rot fungus *Pycnoporus coccineus*, which did not upregulate plant cell wall-degrading enzymes when confronted with competitors (Arfi et al., 2013), indicating they were not important in mediating the interaction.

The GHs upregulated fourfold or more in the interaction zone in this study included one GH 16 in both *G. trabeum* and *R. placenta*, two GH 18 chitinases from *R. placenta*, none of which were likely involved in plant cell wall degradation. Both GH 16 proteins have putative transmembrane regions (Krogh et al., 2001), indicating they may be involved in cell wall reorganization within the fungus. The GH 18 proteins upregulated in *R. placenta* interactions are not transmembrane proteins, and one of them, Pp118230, contains a secretion signal suggesting an extracellular localization. These may play a similar role as GH 18 chitinases in *Phanerochaete chrysosporium* which were differentially expressed during contact with living and dead competitor hyphae, presumably to recycle competitor cell wall components (Karlsson et al., 2016). Laminarinase activity was also higher in the interaction zone than hyphal fronts of single-species cultures (Figure 3), suggesting that branched beta-glucan metabolism is induced in by the interaction.

Plant polysaccharide degrading-enzyme activities were not markedly reduced in the interaction zone compared to equivalent non-interacting hyphae and single species cultures (Figure 3), but these are known to be generally low at the leading hyphal edge of fungal cultures except for pectinase (Zhang et al., 2016; Presley and Schilling, 2017). Pectinase activity was increased in the interaction zone compared to other samples, but no GH 28 pectinases were identified using proteomics analysis or were upregulated at least fourfold compared to non-interacting hyphae (Supplementary Table S1 and Supplementary Data S1). Pectinases in the interaction zone may have gone undetected by our proteomics analysis, as the total number of proteins detected in the interaction was low compared to single-species cultures (Supplementary Table S1; Presley and Schilling, 2017; Presley et al., 2018). Pectinase activity is also performed by some other carbohydrate active enzyme families such as carbohydrate esterase families 8 and 12 and polysaccharide lyase family 1, however, none of these were found to be upregulated in the interaction zone. Furthermore, the polygalacturic acid substrate used to detect pectinase activity in this assay was only guaranteed to be ≥90% (Sigma Aldrich), so some of the activity detected here could be due to the degradation of other cell wall polysaccharides. One way basidiomycetes compete in wood is by capturing more resources than their competitors (Boddy, 2000). Heightened IZ pectinase activity may be an attempt by fungi to “outmaneuver” competitors by overexpressing early stage colonization enzymes including pectinases (Zhang et al., 2016, 2019) and oxidant-producing enzymes (Hori et al., 2014) to facilitate rapid resource capture (Hiscox et al., 2010; Boddy and Hiscox, 2016).

Transcriptomics and proteomics of the interaction zone identified differing strategies for interaction in *G. trabeum* and *R. placenta*. *G. trabeum* appeared to rely more heavily on SM synthesis gene upregulation in response to competitors than *R. placenta* (Figure 4 and Table 2). Interestingly, *G. trabeum* was previously shown to produce pore-forming endotoxin-like proteins in single-species cultures (Presley and Schilling, 2017), but these were not identified in this study and were not upregulated in the interaction. This suggests these putative pore-forming enzymes are not involved in mediating fungal-fungal interactions, but instead may be part of a general anti-herbivory strategy in *G. trabeum* as are homologous proteins in other organisms (Jaquet et al., 1987; Weng et al., 2004). Both fungi upregulated several ORs in the interaction which has been observed previously in interacting hyphae (Arfi et al., 2013). *R. placenta* upregulated more ORs in the IZ than *G. trabeum*, which suggests this species relies more heavily on enzymatic oxidants to facilitate interactions. Xenobiotic-metabolizing ORs are major secretome components in wood-degrading basidiomycetes and some of the oxidoreductases upregulated in the interaction in this study may have similar functional roles (Moody et al., 2018). One OR, Rp105721, was both found exclusively in protein extracts of the interaction (Table 1) and was the 3rd most highly upregulated *R. placenta* gene in the interaction (Tables 1, 2). Rp105721 contains a Rossman-fold NAD(P)-binding domain as well as an FAD-binding domain and shares homology to several other uncharacterized fungal putative FAD-binding proteins. Further characterization of this enzyme may provide insight into interaction mechanisms in this fungus.

Basidiomycetes are rich sources of SMs and several studies have demonstrated the importance of SMs in mediating interspecific interactions in wood decay fungi (Hynes et al., 2007; Evans et al., 2008; Peiris et al., 2008; El Ariebi et al., 2016; O’Leary et al., 2019). *Gloeophyllum* species are known to produce several classes of SMs, including terpenoids (Rasser et al., 2000; Han et al., 2015) and polyketides (Sonnenbichler et al., 1997), that have mild antibacterial and antifungal activity. *G. trabeum* upregulated several SM-synthesis genes in the IZ, implicating the products of these genes in mediating fungal-fungal interactions (Figure 4). Fewer orthologous terpene synthases were also upregulated in *R. placenta* in the IZ, but had generally lower RPKM values than those in *G. trabeum*. In *G. trabeum*, nine upregulated SM-synthesis genes were identified on seven SM-synthesis clusters that were identified using AntiSMASH SM cluster analysis (Figure 5; Weber et al., 2015). Some of these genes, such as the putative orsellinic acid synthase Gt116317, share high homology to characterized SM-synthesizing genes (Lackner et al., 2012, 2013). These SM clusters also contain IZ-upregulated ORs and cytochrome p450s may perform oxidations necessary to produce orsellinic acid-derived metabolites and contribute to the overall greater proportion of ORs upregulated in the IZ (Figure 5). Our study suggests that antimicrobial orsellinic acid-derived SMs may help mediate interspecific interactions in *G. trabeum*, however, this must be confirmed via metabolomics analysis.

Laccases are oxidoreductases that have been widely implicated in basidiomycetes interactions, where they can play a role in melanization (Cordero and Casadevall, 2017) or metabolite detoxification (Hiscox and Boddy, 2017). Brown rot secretomes are generally characterized by a lack of phenol oxidase activity but some do contain these proteins in their genomes (Riley et al., 2014) and produce them in low quantities (Wei et al., 2010). In this study, a laccase in *G. trabeum* was highly upregulated (Gt130426) in the IZ (Table 2), as is seen in protein extracts of the interacting hyphae of several white rot fungi (Baldrian, 2004; Hiscox et al., 2010). However, no laccase activity was found in this test (ABTS assay, data not shown) and no laccase peptides were identified in protein extracts from interaction microcosms, indicating they are not part of the secretome in amounts detectable with our methods. Some secretion signal-containing ascomycete laccases localize to fungal cell walls (Liu and Free, 2016), but Gt130426 does not contain any transmembrane sequences or GPI anchoring sites that would suggest it is localized there.

Basidiomycete interspecific interactions are ubiquitous processes necessary for the survival of these organisms. This study identifies general strategies utilized by wood-degrading basidiomycetes during interspecific combat and identifies interspecific variation in combative mechanisms in two brown rot species. Both species divert resources away from GH and CE production except for pectinase and laminarinase activity, the latter likely to target fungal cell wall β-glucanases. In addition, several orthologous genes were upregulated in the interaction in both species, which suggests an overlap in functionality for these genes which include some cytochrome p450s, proteases, uncharacterized oxidoreductases, hypothetical proteins, terpene synthases and several others. However, there were also patterns unique to each species. *G. trabeum* appeared to exhibit an SM-based interaction strategy by upregulating more SM-synthesis genes than *R. placenta*, while *R. placenta* upregulated a larger proportion of oxidoreductases in the interaction. Several oxidoreductases, including those that could be involved in SM synthesis, metabolite degradation, and cell wall melanization were upregulated in the IZ, indicating they may be important in facilitating interactions. This work provides evidence for the broader functional role of a variety of uncharacterized genes in two model brown rot fungi.

## Data Availability Statement

The raw proteomics data is deposited in the PRIDE/ProteomeXchange database, project accession: PXD009480 and project doi: 10.6019/PXD009480. The raw RPKM sequence data can be found in Supplementary Table S1 and in the Gene Expression Omnibus database (GSE151023).

## Author Contributions

GP generated the cultures, extracted protein, performed proteomics experiment prep, extracted RNA for RNAseq, performed enzyme assays, and wrote the manuscript. JZ performed the RNAseq analysis, data workup for this portion, and edited the manuscript. Ellen Panisko was not listed as an author but she was the technical specialist that performed the LC-MS and data analysis for this portion. SP performed the data analysis for the proteomics. JS was the PI at the time this work was done, directed and funded the research, and edited the manuscript. All authors contributed to the article and approved the submitted version.

## Conflict of Interest

The authors declare that the research was conducted in the absence of any commercial or financial relationships that could be construed as a potential conflict of interest.

## Footnotes

1 https://usegalaxy.org/
