# Supplementary material for: Functional Genomics, Transcriptomics, and Proteomics Reveal Distinct Combat Strategies Between Lineages of Wood-Degrading Fungi With Redundant Wood Decay Mechanisms
Source: Front Microbiol. 2020 Jul 28;11:1646. doi: 10.3389/fmicb.2020.01646 (PMC7399148; doi:10.3389/fmicb.2020.01646)
Supplement: Supplementary file 1 [file Table_1.DOCX]

Table S1: Proteins identified in protein extracts of interacting hyphae of *Gloeophyllum trabeum* and *Rhodonia placenta*.

|  |  |  |  |  |  | RPKM^5^ | | |
| --- | --- | --- | --- | --- | --- | --- | --- | --- |
| Species | ID^1^ | Identification | Swiss prot^2^ | Observations^3^ | Signal P^4^ | IZ | IZ-5Gt/Rp | Gt/Rp_0-5 |
| *G. trabeum* | 101772 | Hypothetical protein | no strong hits | 1 | 25-26 | 372.34 | 31.68 | 8.80 |
| *G. trabeum* | 91843 | Hypothetical protein | no strong hits | 1 | no | 130.13 | 371.62 | 261.53 |
| *G. trabeum* | 128123 | Hypothetical protein | no strong hits | 1 | no | 6.39 | 19.34 | 19.93 |
| *G. trabeum* | 108762 | Hypothetical protein | no strong hits | 7 | 17-18 | 372.56 | 93.60 | 24.68 |
| *G. trabeum* | 81033 | Metallophosphatase | metallophosphatase | 1 | no | 30.83 | 81.98 | 47.17 |
| *G. trabeum* | 136352 | Protease, Acid | Acid protease | 4 | 19-20 | 23.35 | 78.29 | 41.56 |
| *G. trabeum* | 112815 | Ribosomal protein S15 | Ribosomal S37 | 1 | no | 54.30 | 94.75 | 89.84 |
| *G. trabeum* | 116675 | Formamidase | Formamidase | 6 | no | 69.43 | 259.70 | 137.34 |
| *G. trabeum* | 121308 | GH 115 | no strong hits | 8 | 21-22 | 145.84 | 1195.17 | 286.65 |
| *G. trabeum* | 69843 | GH 3 | β-glucosidase | 7 | 18-19 | 34.34 | 1218.55 | 151.98 |
| *G. trabeum* | 130688 | Hypothetical protein | no strong hits | 11 | 16-17 | 0.00 | 4.49 | 0.00 |
| *G. trabeum* | 44870 | Hypothetical protein | no strong hits | 17 | 21-22 | 721.56 | 16.89 | 3.95 |
| *G. trabeum* | 112240 | Hypothetical protein | no strong hits | 2 | 17-18 | 254.59 | 755.71 | 342.62 |
| *G. trabeum* | 112664 | Hypothetical protein | no strong hits | 39 | no | 158.81 | 1563.02 | 51.47 |
| *G. trabeum* | 92695 | Hypothetical protein | no strong hits | 4 | 19-20 | 3599.50 | 12.76 | 8.82 |
| *G. trabeum* | 116097 | Hypothetical protein | no strong hits | 5 | 19-20 | 55.10 | 291.37 | 106.48 |
| *G. trabeum* | 93553 | Hypothetical protein | no strong hits | 1 | no | 73.20 | 130.40 | 19.68 |
| *G. trabeum* | 138715 | Hypothetical protein | no strong hits | 10 | no | 2560.24 | 8381.62 | 931.09 |
| *G. trabeum* | 129354 | Hypothetical protein | no strong hits | 2 | 19-20 | 587.58 | 40.36 | 11.31 |
| *G. trabeum* | 61365 | Hypothetical protein | no strong hits | 6 | no | 2227.33 | 1133.11 | 7464.69 |
| *G. trabeum* | 104432 | Nuclear transport factor | Nuclear transport factor | 2 | no | 224.25 | 849.69 | 346.95 |
| *G. trabeum* | 62581 | Protease, Acid | Aspergillopepsin | 46 | 18-19 | 371.90 | 3759.26 | 1024.04 |
| *G. trabeum* | 48672 | Protease, Tripeptidyl | Tripeptidyl Protease | 5 | 17-18 | 18.20 | 1233.41 | 97.90 |
| *G. trabeum* | 39789 | Protease, Tripeptidyl | Tripeptidyl Protease | 11 | no | 121.48 | 400.82 | 338.67 |
| *G. trabeum* | 134939 | Thioredoxin | Thioredoxin | 1 | no | 392.78 | 1995.75 | 304.36 |
| *P. placenta* | 1183565 | AB hydrolase | Lipase | 2 | no | #N/A | #N/A | #N/A |
| *P. placenta* | 105721 | FAD-oxidoreductase | FAD binding protein | 4 | 18-19 | 257.88 | 1.32 | 1.53 |
| *P. placenta* | 92184 | Hypothetical protein | no strong hits | 1 | no | 8.22 | 19.23 | 12.17 |
| *P. placenta* | 99255 | Hypothetical protein | no strong hits | 7 | no | 0.41 | 0.46 | 0.95 |
| *P. placenta* | 93455 | Hypothetical protein | no strong hits | 1 | no | 2.33 | 3.46 | 2.03 |
| *P. placenta* | 98779 | Hypothetical protein | no strong hits | 11 | no | 5.34 | 14.21 | 5.20 |
| *P. placenta* | 101552 | Hypothetical protein | no strong hits | 4 | 16-17 | 4.19 | 11.53 | 1.90 |
| *P. placenta* | 96562 | Hypothetical protein | no strong hits | 36 | 16-17 | 0.69 | 0.12 | 0.35 |
| *P. placenta* | 1127001 | Protease, Acid | Polyporopepsin | 3 | 19-20 | #N/A | #N/A | #N/A |

^1^Protein ID numbers from the DOE JGI Mycocosm database (Grigoriev et al., 2014).

^2^Putative functions determined by BLAST searches of SWISS PROT database (Bateman et al., 2015).

^3^Number of observations (spectral counts)

^4^Secretion signals were identified using Signal P prediction server (Armenteros et al., 2019).

^5^RPKM values from RNAseq data from interaction microcosms and single species cultures.

Table S2: Proteins identified in protein extracts of *Gloeophyllum trabeum* hyphae 5 mm outside of the interaction zone (IZ-5Gt). Proteins are listed with the total number of observations.

|  |  |  |  |  |  | RPKM^5^ | | |
| --- | --- | --- | --- | --- | --- | --- | --- | --- |
| Species | ID^1^ | Name | Swiss prot^2^ | Observations^3^ | Signal P^4^ | IZ | IZ-5Gt | Gt_0-5 |
| *G. trabeum* | 36245 | Amidase | Amidase | 2 | 21-22 | 13.42 | 89.50 | 21.35 |
| *G. trabeum* | 46347 | CE 15 | Glucuronly esterase | 2 | 19-20 | #N/A | #N/A | #N/A |
| *G. trabeum* | 138785 | GH 10 | endo-β-1,4-xylanase | 1 | 19-20 | 58.55 | 1622.82 | 141.77 |
| *G. trabeum* | 121308 | GH 115 | no strong hits | 55 | 21-22 | 145.84 | 1195.17 | 286.65 |
| *G. trabeum* | 138821 | GH 12 | Xyloglucan specific endoglucanase | 2 | 20-21 | 38.60 | 2681.53 | 630.53 |
| *G. trabeum* | 69843 | GH 3 | β-glucosidase | 34 | 18-19 | 34.34 | 1218.55 | 151.98 |
| *G. trabeum* | 122002 | GH 3 | endo-β-xylosidase | 14 | 21-22 | 174.11 | 1894.37 | 362.08 |
| *G. trabeum* | 63180 | GH 5 | Endoglucanase | 6 | 20-21 | 372.18 | 1111.49 | 925.02 |
| *G. trabeum* | 81248 | Hypothetical | no strong hits | 5 | 25-26 | 236.27 | 2201.28 | 699.19 |
| *G. trabeum* | 139330 | Hypothetical | no strong hits | 1 | no | 14.46 | 20.12 | 7.08 |
| *G. trabeum* | 65283 | Hypothetical protein | no strong hits | 26 | 18-19 | 244.01 | 3468.86 | 1239.35 |
| *G. trabeum* | 48343 | Hypothetical protein | carboxylesterase | 10 | 21-22 | 35.50 | 211.31 | 51.92 |
| *G. trabeum* | 96567 | Hypothetical protein | Carboxylestrease | 8 | 18-19 | 39.86 | 665.45 | 120.50 |
| *G. trabeum* | 131166 | Hypothetical protein | no strong hits | 4 | no | 0.00 | 0.00 | 0.00 |
| *G. trabeum* | 122332 | Hypothetical protein | no strong hits | 1 | 23-24 | 181.51 | 2645.22 | 285.34 |
| *G. trabeum* | 131837 | Hypothetical protein | Hercynyl cysteine sulfoxide lyase | 1 | no | 68.17 | 58.62 | 60.87 |
| *G. trabeum* | 122240 | Hypothetical protein | no strong hits | 1 | 17-18 | #N/A | #N/A | #N/A |
| *G. trabeum* | 96019 | Hypothetical protein | no strong hits | 1 | no | 0.02 | 0.19 | 0.12 |
| *G. trabeum* | 48407 | Hypothetical protein | no strong hits | 1 | no | 27.15 | 99.60 | 45.88 |
| *G. trabeum* | 112674 | Hypothetical protein | Transcription factor | 1 | no | 18.48 | 33.97 | 29.32 |
| *G. trabeum* | 36877 | Hypothetical protein | no strong hits | 1 | no | 10.11 | 22.86 | 13.00 |
| *G. trabeum* | 73954 | Hypothetical protein | no strong hits | 1 | no | 5.53 | 7.56 | 4.64 |
| *G. trabeum* | 93128 | Hypothetical protein | no strong hits | 1 | no | 0.66 | 1.02 | 0.89 |
| *G. trabeum* | 124440 | Hypothetical protein | microtubule binding protein | 1 | no | #N/A | #N/A | #N/A |
| *G. trabeum* | 138715 | Hypothetical protein | no strong hits | 1 | no | 2560.24 | 8381.62 | 931.09 |
| *G. trabeum* | 24740 | Lipase | Lipase | 1 | no | 23.77 | 234.05 | 64.17 |
| *G. trabeum* | 130556 | Oxalate decarboxylase | Oxalate decarboxylase | 1 | 19-20 | 28.43 | 1574.42 | 106.53 |
| *G. trabeum* | 81017 | Profilin | Profilin | 1 | no | 287.57 | 1695.80 | 375.05 |
| *G. trabeum* | 62581 | Protease, Acid | Aspergillopepsin | 50 | 18-19 | 371.90 | 3759.26 | 1024.04 |
| *G. trabeum* | 57888 | Protease, Acid | Polyporopepsin | 6 | 19-20 | 241.59 | 3515.21 | 526.83 |
| *G. trabeum* | 48672 | Protease, Tripeptidyl | Tripeptidyl Protease | 29 | 17-18 | 18.20 | 1233.41 | 97.90 |
| *G. trabeum* | 48851 | Protease, Tripeptidyl | Tripeptidyl Protease | 4 | 17-18 | 16.01 | 74.45 | 21.68 |
| *G. trabeum* | 39789 | Protease, Tripeptidyl | Tripeptidyl Protease | 2 | no | 121.48 | 400.82 | 338.67 |

^1^Protein ID numbers from the DOE JGI Mycocosm database (Grigoriev et al., 2014).

^2^Putative functions determined by BLAST searches of SWISS PROT database (Bateman et al., 2015).

^3^Number of observations (spectral counts)

^4^Secretion signals were identified using Signal P prediction server (Armenteros et al., 2019).

^5^RPKM values from RNAseq data from interaction microcosms and single species cultures.

Table S3: Proteins identified in protein extracts of *Rhodonia placenta* hyphae 5 mm outside of the interaction zone (IZ-5Rp).

|  |  |  |  |  |  | RPKM^5^ | | |
| --- | --- | --- | --- | --- | --- | --- | --- | --- |
| Species | ID^1^ | Name | Swiss prot^2^ | Observations^3^ | Signal P^4^ | IZ | IZ-5Rp | Rp_0-5 |
| *P. placenta* | 127015 | Cholinesterase | Fatty acyl coA hydrolase | 2 | 26-27 | 71.00 | 518.75 | 172.71 |
| *P. placenta* | 127015 | Cholinesterase | Paranitro benzylesterase | 19 | 26-27 | 71.00 | 518.75 | 172.71 |
| *P. placenta* | 96457 | DNA ligase | DNA ligase | 1 | no | 1.62 | 3.57 | 4.60 |
| *P. placenta* | 1076117 | GH 115 | no strong hits | 1 | 19-20 | #N/A | #N/A | #N/A |
| *P. placenta* | 57564 | GH 2 | β-mannosidase | 12 | no | 57.33 | 1696.72 | 181.55 |
| *P. placenta* | 134890 | GH 3 | exo-β-1,4-xylosidase | 1 | 18-19 | #N/A | #N/A | #N/A |
| *P. placenta* | 107557 | GH 3 | β-glucosidase | 5 | 24-25 | 182.14 | 4240.92 | 570.31 |
| *P. placenta* | 1073356 | Glutamate synthase | Glutamate synthase | 1 | no | #N/A | #N/A | #N/A |
| *P. placenta* | 104279 | Hypothetical protein | no strong hits | 1 | no | 1.39 | 1.91 | 1.98 |
| *P. placenta* | 99255 | Hypothetical protein | no strong hits | 13 | no | 0.41 | 0.46 | 0.95 |
| *P. placenta* | 1064989 | Hypothetical protein | Pumilio domain protein | 2 | no | #N/A | #N/A | #N/A |
| *P. placenta* | 100575 | Hypothetical protein | no strong hits | 1 | no | 0.24 | 0.11 | 0.47 |
| *P. placenta* | 101774 | Hypothetical protein | no strong hits | 2 | no | 47.40 | 19.58 | 111.79 |
| *P. placenta* | 90946 | Hypothetical protein | no strong hits | 5 | 17-18 | 4.84 | 527.94 | 19.41 |
| *P. placenta* | 1141136 | Peptidase, Acid | Aspergillopepsin | 3 | 18-19 | #N/A | #N/A | #N/A |

^1^Protein ID numbers from the DOE JGI Mycocosm database (Grigoriev et al., 2014).

^2^Putative functions determined by BLAST searches of SWISS PROT database (Bateman et al., 2015).

^3^Number of observations (spectral counts)

^4^Secretion signals were identified using Signal P prediction server (Armenteros et al., 2019).

^5^RPKM values from RNAseq data from interaction microcosms and single species cultures.

Table S4: Glycoside hydrolases and carbohydrate esterases found in secretomes of *Gloeophyllum trabeum* on aspen wafers and their expression levels in the interaction zone (IZ) and at the actively growing hyphal front of *G. trabeum* cultures (Gt_0-5).

| ID^1^ | Name^2^ | Swiss prot^3^ | RPKM IZ | RPKM Gt_0-5 | Log2 (IZ/Gt_0-5) | p value | q value |
| --- | --- | --- | --- | --- | --- | --- | --- |
| 117128 | CE 1 | Acetyl xylan esterase | 72.84 | 198.89 | -1.45 | 0.09 | 0.16 |
| 46545 | CE 15 | 4-O-methyl-glucuronyl methylesterase | 10.75 | 43.71 | -2.02 | 0.02 | 0.04 |
| 48624 | CE 16 | Uncharacterized CE 16 | 52.50 | 323.17 | -2.62 | 0.03 | 0.05 |
| 56205 | CE 16 | Unchracterized CE 16 | 214.59 | 374.05 | -0.80 | 0.33 | 0.42 |
| 32318 | CE 4 | Chitin deacetylase | 602.75 | 722.37 | -0.26 | 0.75 | 0.80 |
| 112531 | CE 8 | Pectinesterase | 289.92 | 543.26 | -0.91 | 0.28 | 0.36 |
| 140289 | GH 10 | Endo-β-1,4-xylanase | 53.10 | 98.73 | -0.89 | 0.28 | 0.36 |
| 46499 | GH 10 | Endo-β-1,4-xylanase | 286.22 | 697.95 | -1.29 | 0.15 | 0.22 |
| 121307 | GH 115 | no strong hits | 208.56 | 433.95 | -1.06 | 0.19 | 0.26 |
| 121308 | GH 115 | no strong hits | 145.84 | 286.65 | -0.97 | 0.26 | 0.34 |
| 138821 | GH 12 | Xyloglucan specific endo-β-1,4-glucanase | 38.60 | 630.53 | -4.03 | 0.00 | 0.00 |
| 61700 | GH 15- CBM 20 | Glucoamylase, CBM20 domain | 50.64 | 76.25 | -0.59 | 0.49 | 0.57 |
| 52752 | GH 16 | β-glucan synthesis protein | 64.22 | 104.35 | -0.70 | 0.39 | 0.47 |
| 122074 | GH 18 | Chitinase | 52.40 | 127.36 | -1.28 | 0.14 | 0.20 |
| 115191 | GH 2 | β-Mannanase | 112.82 | 282.05 | -1.32 | 0.11 | 0.18 |
| 116582 | GH 20 | β-N-acetylglucosaminidase | 179.72 | 454.79 | -1.34 | 0.14 | 0.21 |
| 77583 | GH 20 | β-N-acetylglucosaminidase | 36.15 | 92.35 | -1.35 | 0.14 | 0.20 |
| 117566 | GH 27 | α-galactosidase | 526.99 | 760.44 | -0.53 | 0.53 | 0.60 |
| 110574 | GH 28 | Pectinase | 268.53 | 784.22 | -1.55 | 0.06 | 0.10 |
| 120615 | GH 28 | Pectinase | 70.38 | 153.87 | -1.13 | 0.21 | 0.28 |
| 138836 | GH 28 | Pectinase | 121.24 | 291.71 | -1.27 | 0.12 | 0.19 |
| 6650 | GH 28 | Pectinase | 77.29 | 212.37 | -1.46 | 0.08 | 0.14 |
| 69843 | GH 3 | β-glucosidase | 34.34 | 151.98 | -2.15 | 0.07 | 0.13 |
| 75899 | GH 3 | β-glucosidase | 172.95 | 322.48 | -0.90 | 0.30 | 0.39 |
| 122002 | GH 3 | Exo-β-1,4-xylosidase | 174.11 | 362.08 | -1.06 | 0.22 | 0.30 |
| 141329 | GH 31 | α-xylosidase | 143.59 | 271.27 | -0.92 | 0.27 | 0.35 |
| 111095 | GH 35 | β-galactosidase | 100.18 | 188.55 | -0.91 | 0.28 | 0.36 |
| 112205 | GH 47 | Mannosyl oligosaccaride α-1,2-mannosidase | 323.39 | 696.63 | -1.11 | 0.19 | 0.26 |
| 114574 | GH 5 | Endo-β-1,4-mannanase | 144.44 | 107.21 | 0.43 | 0.63 | 0.70 |
| 110405 | GH 5 | Endo-β-1,4-mannanase | 207.09 | 316.83 | -0.61 | 0.51 | 0.59 |
| 135369 | GH 5 | Endo-β-1,4-mannanase | 154.67 | 393.86 | -1.35 | 0.21 | 0.29 |
| 63180 | GH 5 | Endo-β-1,4-glucanase | 372.18 | 925.02 | -1.31 | 0.22 | 0.30 |
| 134804 | GH 51 | α-arabinofuranosidase | 27.16 | 73.57 | -1.44 | 0.10 | 0.16 |
| 126879 | GH 55 | Exo-β-1,3-glucosidase | 181.07 | 291.93 | -0.69 | 0.52 | 0.60 |
| 113553 | GH 72-CBM 43 | β-1,3-glucanosyltransferase | 195.01 | 338.41 | -0.80 | 0.37 | 0.46 |
| 116837 | GH 79 | β-glucuronidase | 49.42 | 116.63 | -1.24 | 0.17 | 0.25 |
| 81814 | GH 92 | Glycosidase | 40.51 | 68.76 | -0.76 | 0.38 | 0.47 |

^1^Protein ID numbers from the DOE JGI Mycocosm database (Grigoriev et al., 2014), alleles are listed in red italics under its pair.

^2^Carbohydrate esterase (CE) and glycoside hydrolase (GH) families as defined in the CAZy database (Lombard et al., 2014).

^3^Putative functions determined by BLAST searches of SWISS PROT database (Bateman et al., 2015).

Table S5: Glycoside hydrolases and carbohydrate esterases found in secretomes of *Gloeophyllum trabeum* on aspen wafers and their expression levels in the interaction zone (IZ) and in *G. trabeum* hyphae 5 mm outside the interaction (IZ-5Gt).

| ID^1^ | Name^2^ | Swiss prot^3^ | RPKM IZ-5Gt | RPKM IZ | Log_2_(IZ/IZ-5Gt) | p value | q value |
| --- | --- | --- | --- | --- | --- | --- | --- |
| 117128 | CE 1 | Acetyl xylan esterase | 2269.23 | 72.84 | -4.96 | 0.00 | 0.00 |
| 46545 | CE 15 | 4-O-methyl-glucuronyl methylesterase | 206.39 | 10.75 | -4.26 | 0.00 | 0.00 |
| 48624 | CE 16 | Uncharacterized CE 16 | 1859.85 | 52.50 | -5.15 | 0.00 | 0.00 |
| 56205 | CE 16 | Unchracterized CE 16 | 2522.71 | 214.59 | -3.56 | 0.00 | 0.00 |
| 32318 | CE 4 | Chitin deacetylase | 1283.74 | 602.75 | -1.09 | 0.25 | 0.33 |
| 112531 | CE 8 | Pectinesterase | 954.52 | 289.92 | -1.72 | 0.06 | 0.10 |
| 140289 | GH 10 | Endo-β-1,4-xylanase | 201.13 | 53.10 | -1.92 | 0.10 | 0.16 |
| 46499 | GH 10 | Endo-β-1,4-xylanase | 3116.71 | 286.22 | -3.44 | 0.00 | 0.00 |
| 121307 | GH 115 | no strong hits | 189.00 | 208.56 | 0.14 | 0.87 | 0.90 |
| 121308 | GH 115 | no strong hits | 1195.17 | 145.84 | -3.03 | 0.00 | 0.00 |
| 138821 | GH 12 | Xyloglucan specific endo-β-1,4-glucanase | 2681.53 | 38.60 | -6.12 | 0.00 | 0.00 |
| 61700 | GH 15- CBM 20 | Glucoamylase, CBM20 domain | 133.45 | 50.64 | -1.40 | 0.15 | 0.22 |
| 52752 | GH 16 | β-glucan synthesis protein | 154.04 | 64.22 | -1.26 | 0.23 | 0.31 |
| 122074 | GH 18 | Chitinase | 301.81 | 52.40 | -2.53 | 0.01 | 0.01 |
| 115191 | GH 2 | β-Mannanase | 671.19 | 112.82 | -2.57 | 0.01 | 0.01 |
| 116582 | GH 20 | β-N-acetylglucosaminidase | 1142.88 | 179.72 | -2.67 | 0.01 | 0.02 |
| 77583 | GH 20 | β-N-acetylglucosaminidase | 89.44 | 36.15 | -1.31 | 0.19 | 0.27 |
| 117566 | GH 27 | α-galactosidase | 2774.45 | 526.99 | -2.40 | 0.01 | 0.02 |
| 110574 | GH 28 | Pectinase | 985.55 | 268.53 | -1.88 | 0.04 | 0.06 |
| 120615 | GH 28 | Pectinase | 146.11 | 70.38 | -1.05 | 0.47 | 0.55 |
| 138836 | GH 28 | Pectinase | 867.03 | 121.24 | -2.84 | 0.00 | 0.01 |
| 6650 | GH 28 | Pectinase | 263.06 | 77.29 | -1.77 | 0.07 | 0.12 |
| 69843 | GH 3 | β-glucosidase | 1218.55 | 34.34 | -5.15 | 0.00 | 0.01 |
| 75899 | GH 3 | β-glucosidase | 1495.82 | 172.95 | -3.11 | 0.02 | 0.04 |
| 122002 | GH 3 | Exo-β-1,4-xylosidase | 1894.37 | 174.11 | -3.44 | 0.00 | 0.00 |
| 141329 | GH 31 | α-xylosidase | 552.41 | 143.59 | -1.94 | 0.04 | 0.07 |
| 111095 | GH 35 | β-galactosidase | 283.96 | 100.18 | -1.50 | 0.12 | 0.19 |
| 112205 | GH 47 | α-1,2-mannosidase | 1482.78 | 323.39 | -2.20 | 0.02 | 0.04 |
| 114574 | GH 5 | Endo-β-1,4-mannanase | 296.39 | 144.44 | -1.04 | 0.30 | 0.38 |
| 110405 | GH 5 | Endo-β-1,4-mannanase | 2361.21 | 207.09 | -3.51 | 0.00 | 0.01 |
| 135369 | GH 5 | Endo-β-1,4-mannanase | 2305.82 | 154.67 | -3.90 | 0.01 | 0.02 |
| 63180 | GH 5 | Endo-β-1,4-glucanase | 1111.49 | 372.18 | -1.58 | 0.18 | 0.25 |
| 134804 | GH 51 | α-arabinofuranosidase | 163.91 | 27.16 | -2.59 | 0.01 | 0.02 |
| 126879 | GH 55 | Exo-β-1,3-glucosidase | 728.58 | 181.07 | -2.01 | 0.13 | 0.20 |
| 113553 | GH 72-CBM43 | β-1,3-glucanosyltransferase | 925.82 | 195.01 | -2.25 | 0.03 | 0.05 |
| 116837 | GH 79 | β-glucuronidase | 453.76 | 49.42 | -3.20 | 0.00 | 0.00 |
| 81814 | GH 92 | Glycosidase | 197.26 | 40.51 | -2.28 | 0.02 | 0.04 |

^1^Protein ID numbers from the DOE JGI Mycocosm database (Grigoriev et al., 2014), alleles are listed in red italics under its pair.

^2^Carbohydrate esterase (CE) and glycoside hydrolase (GH) families as defined in the CAZy database (Lombard et al., 2014).

^3^Putative functions determined by BLAST searches of SWISS PROT database (Bateman et al., 2015).

Table S6: Glycoside hydrolases and carbohydrate esterases found in secretomes of *Rhodonia placenta* on aspen wafers and their expression levels in the interaction zone (IZ) and at the actively growing hyphal front of *R. placenta* cultures (Rp_0-5).

| Protein ID^1^ | Name^2^ | Swiss prot^3^ | RPKM IZ | RPKM Rp_0-5 | Log_2_(IZ/Rp_0-5) | p value | q value |
| --- | --- | --- | --- | --- | --- | --- | --- |
| 20448 | CE 10 | Lipase | 3.06 | 7.54 | -1.30 | 0.20 | 0.28 |
| *50078* | CE 10 | Lipase | 3.64 | 5.91 | -0.70 | 0.47 | 0.56 |
| 125801 | CE 16 | Carboxylesterase | 197.41 | 636.49 | -1.69 | 0.11 | 0.18 |
| 108959 | CE 16 | Carboxylesterase | 53.32 | 252.08 | -2.24 | 0.06 | 0.10 |
| 106710 | CE 16 | no strong hits | 100.26 | 357.77 | -1.84 | 0.09 | 0.15 |
| 113670 | GH 10 | Endo-β-1,4-xylanase | 22.07 | 53.37 | -1.27 | 0.21 | 0.29 |
| 112658 | GH 12 | Endo-β-1,4-glucanase | 7.29 | 119.36 | -4.03 | 0.00 | 0.00 |
| *121191* | GH 12 | Endo-β-1,4-glucanase | 20.47 | 215.79 | -3.40 | 0.01 | 0.03 |
| 113112 | GH 15 | Glucoamylase | 32.63 | 43.73 | -0.42 | 0.68 | 0.74 |
| *117345* | GH 15 | Glucoamylase | 31.36 | 45.21 | -0.53 | 0.59 | 0.67 |
| 116903 | GH 16 | Endo-β-1,3(4)-glucanase | 114.92 | 103.07 | 0.16 | 0.88 | 0.91 |
| *128334* | GH 16 | Endo-β-1,3(4)-glucanase | 118.65 | 114.80 | 0.05 | 0.96 | 0.97 |
| 112941 | GH 16 | Endo-β-1,3(4)-glucanase | 173.55 | 193.81 | -0.16 | 0.87 | 0.90 |
| *61809* | GH 16 | Endo-β-1,3(4)-glucanase | 60.02 | 33.25 | 0.85 | 0.37 | 0.46 |
| 54949 | GH 16 | β-glucan synthesis protein | 7.50 | 15.15 | -1.01 | 0.34 | 0.42 |
| 44128 | GH 18 | Chitinase | 13.21 | 12.84 | 0.04 | 0.96 | 0.97 |
| *53332* | GH 18 | Chitinase | 17.78 | 15.64 | 0.18 | 0.88 | 0.90 |
| 128150 | GH 27 | α-galactosidase | 54.98 | 165.15 | -1.59 | 0.14 | 0.21 |
| *98662* | GH 27 | α-galactosidase | 96.59 | 245.62 | -1.35 | 0.20 | 0.28 |
| 111730 | GH 28 | Pectinase | 10.46 | 293.31 | -4.81 | 0.00 | 0.00 |
| *43189* | GH 28 | Pectinase | 165.51 | 1311.10 | -2.99 | 0.01 | 0.03 |
| 107557 | GH 3 | β-glucosidase | 182.14 | 570.31 | -1.65 | 0.21 | 0.29 |
| 115593 | GH 47 | α-1,2-mannosidase | 75.87 | 284.80 | -1.91 | 0.07 | 0.13 |
| *46679* | GH 47 | α-1,2-mannosidase | 67.49 | 263.88 | -1.97 | 0.07 | 0.12 |
| 103675 | GH 5 | Endoglucanase | 54.26 | 237.64 | -2.13 | 0.05 | 0.09 |
| *117690* | GH 5 | Endoglucanase | 55.24 | 215.47 | -1.96 | 0.07 | 0.13 |
| 95568 | GH 5 | Endo-β-1,4-mannosidase | 68.71 | 259.93 | -1.92 | 0.08 | 0.13 |
| 115648 | GH 5 | Endoglucanase | 409.80 | 728.26 | -0.83 | 0.40 | 0.48 |
| *108962* | GH 5 | Endoglucanase | 17.79 | 63.00 | -1.82 | 0.09 | 0.15 |
| 121831 | GH 5 | Endo-β-1,4-mannanase | 172.17 | 393.80 | -1.19 | 0.24 | 0.32 |
| *134772* | GH 5 | Endo-β-1,4-mannanase | #N/A | #N/A | #N/A | #N/A | #N/A |
| 100251 | GH 51 | α-arabinofuranosidase | 31.65 | 69.74 | -1.14 | 0.28 | 0.36 |
| 108648 | GH 55 | Exo-β-1,3-glucanase | 40.74 | 50.43 | -0.31 | 0.81 | 0.85 |
| 105490 | GH 55 | Endo-β-1,3-glucanase | 0.36 | 0.38 | -0.09 | 1.00 | 1.00 |
| 119394 | GH 55 | Exo-β-1,3-glucanase | 93.97 | 30.67 | 1.62 | 0.11 | 0.18 |
| 111332 | GH 79 | β-glucuronidase | 27.56 | 95.77 | -1.80 | 0.09 | 0.16 |

^1^Protein ID numbers from the DOE JGI Mycocosm database (Grigoriev et al., 2014), alleles are listed in red italics under its pair.

^2^Carbohydrate esterase (CE) and glycoside hydrolase (GH) families as defined in the CAZy database (Lombard et al., 2014).

^3^Putative functions determined by BLAST searches of SWISS PROT database (Bateman et al., 2015).

Table S7: Glycoside hydrolases and carbohydrate esterases found in secretomes of *Rhodonia placenta* on aspen wafers and their expression levels in the interaction zone (IZ) and in *R. placenta* hyphae 5 mm outside the interaction (IZ-5Rp).

| Protein ID^1^ | Name^2^ | Swiss prot^3^ | RPKM IZ | RPKM Pp-5 | Log_2_(IZ/IZ-5Rp) | p_value | q_value |
| --- | --- | --- | --- | --- | --- | --- | --- |
| 20448 | CE 10 | Lipase | 3.06 | 58.29 | -4.25 | 0.01 | 0.01 |
| *50078* | CE 10 | Lipase | 3.64 | 71.78 | -4.30 | 0.00 | 0.01 |
| 125801 | CE 16 | Carboxylesterase | 197.41 | 5785.33 | -4.87 | 0.00 | 0.01 |
| 108959 | CE 16 | Carboxylesterase | 53.32 | 2245.00 | -5.40 | 0.00 | 0.00 |
| 106710 | CE 16 | No strong hits | 100.26 | 3209.47 | -5.00 | 0.00 | 0.00 |
| 113670 | GH 10 | Endo-β-1,4-xylanase | 22.07 | 764.04 | -5.11 | 0.00 | 0.00 |
| 112658 | GH 12 | Endo-β-1,4-glucanase | 7.29 | 645.32 | -6.47 | 0.00 | 0.00 |
| *121191* | GH 12 | Endo-β-1,4-glucanase | 20.47 | 1491.89 | -6.19 | 0.00 | 0.00 |
| 113112 | GH 15 | Glucoamylase | 32.63 | 255.57 | -2.97 | 0.03 | 0.06 |
| *117345* | GH 15 | Glucoamylase | 31.36 | 67.80 | -1.11 | 0.40 | 0.48 |
| 116903 | GH 16 | Endo-β-1,3(4)-glucanase | 114.92 | 287.28 | -1.32 | 0.33 | 0.42 |
| *128334* | GH 16 | Endo-β-1,3(4)-glucanase | 118.65 | 540.79 | -2.19 | 0.11 | 0.18 |
| 112941 | GH 16 | Endo-β-1,3(4)-glucanase | 173.55 | 915.19 | -2.40 | 0.06 | 0.11 |
| *61809* | GH 16 | Endo-β-1,3(4)-glucanase | 60.02 | 385.68 | -2.68 | 0.04 | 0.08 |
| 54949 | GH 16 | β-glucan synthesis protein | 7.50 | 124.87 | -4.06 | 0.01 | 0.02 |
| 44128 | GH 18 | Chitinase | 13.21 | 188.40 | -3.83 | 0.01 | 0.02 |
| *53332* | GH 18 | Chitinase | 17.78 | 222.48 | -3.65 | 0.01 | 0.02 |
| 128150 | GH 27 | α-galactosidase | 54.98 | 1032.60 | -4.23 | 0.01 | 0.01 |
| *98662* | GH 27 | α-galactosidase | 96.59 | 1921.19 | -4.31 | 0.00 | 0.01 |
| 111730 | GH 28 | Pectinase | 10.46 | 4.29 | 1.29 | 0.31 | 0.40 |
| *43189* | GH 28 | Pectinase | 165.51 | 83.91 | 0.98 | 0.48 | 0.56 |
| 107557 | GH 3 | β-glucosidase | 182.14 | 4240.92 | -4.54 | 0.00 | 0.00 |
| 115593 | GH 47 | α-1,2-mannosidase | 75.87 | 455.87 | -2.59 | 0.05 | 0.09 |
| *46679* | GH 47 | α-1,2-mannosidase | 67.49 | 360.79 | -2.42 | 0.07 | 0.12 |
| 103675 | GH 5 | Endoglucanase | 54.26 | 2550.33 | -5.55 | 0.00 | 0.00 |
| *117690* | GH 5 | Endoglucanase | 55.24 | 1781.16 | -5.01 | 0.00 | 0.00 |
| 95568 | GH 5 | Endo-β-1,4-mannosidase | 68.71 | 2024.03 | -4.88 | 0.00 | 0.00 |
| 115648 | GH 5 | Endoglucanase | 409.80 | 13777.20 | -5.07 | 0.00 | 0.00 |
| *108962* | GH 5 | Endoglucanase | 17.79 | 559.44 | -4.97 | 0.00 | 0.00 |
| 121831 | GH 5 | Endo-β-1,4-mannanase | 172.17 | 3451.12 | -4.33 | 0.00 | 0.01 |
| *134772* | GH 5 | Endo-β-1,4-mannanase | #N/A | #N/A | #N/A | #N/A | #N/A |
| 100251 | GH 51 | α-arabinofuranosidase | 31.65 | 283.94 | -3.17 | 0.03 | 0.05 |
| 108648 | GH 55 | Exo-β-1,3-glucanase | 40.74 | 297.09 | -2.87 | 0.03 | 0.06 |
| 105490 | GH 55 | Endo-β-1,3-glucanase | 0.36 | 6.07 | -4.07 | 0.01 | 0.02 |
| 119394 | GH 55 | Exo-β-1,3-glucanase | 93.97 | 455.51 | -2.28 | 0.08 | 0.13 |
| 111332 | GH 79 | β-glucuronidase | 27.56 | 1184.16 | -5.43 | 0.00 | 0.00 |

^1^Protein ID numbers from the DOE JGI Mycocosm database (Grigoriev et al., 2014), alleles are listed in red italics under its pair.

^2^Carbohydrate esterase (CE) and glycoside hydrolase (GH) families as defined in the CAZy database (Lombard et al., 2014).

^3^Putative functions determined by BLAST searches of SWISS PROT database (Bateman et al., 2015).

References

Armenteros, J. J. A., Tsirigos, K. D., Sonderby, C. K., Petersen, T. N., Winther, O., Brunak, S., et al. (2019). SignalP 5.0 improves signal peptide predictions using deep neural networks. *Nat. Biotechnol.* 37, 420+. doi:10.1038/s41587-019-0036-z.

Bateman, A., Martin, M. J., O’Donovan, C., Magrane, M., Apweiler, R., Alpi, E., et al. (2015). UniProt: A hub for protein information. *Nucleic Acids Res.* 43, D204–D212. doi:10.1093/nar/gku989.

Grigoriev, I. V, Nikitin, R., Haridas, S., Kuo, A., Ohm, R., Otillar, R., et al. (2014). MycoCosm portal: Gearing up for 1000 fungal genomes. *Nucleic Acids Res.* 42, D699–D704. doi:10.1093/nar/gkt1183.

Lombard, V., Ramulu, H. G., Drula, E., Coutinho, P. M., and Henrissat, B. (2014). The carbohydrate-active enzymes database (CAZy) in 2013. *Nucleic Acids Res.* 42, D490–D495. doi:10.1093/nar/gkt1178.
